# Synthesis and Phase Evolution of Ultra-High Temperature MC-Type Carbides (M = Hf, Ta, Nb, Zr, Ti) via a Molecular Precursor Approach

**DOI:** 10.3390/molecules31183193

**Published:** 2026-09-10

**Authors:** Junyi Zheng, Haiyun Peng, Xiantao Yang, Yuenong Liu, Zhaoju Yu

**Affiliations:** 1 Key Laboratory of High Performance Ceramic Fibers, Ministry of Education, College of Materials, Xiamen University, Xiamen 361005, China; 20720241150188@stu.xmu.edu.cn (J.Z.); 20720241150146@stu.xmu.edu.cn (H.P.); xiantaoyang@stu.xmu.edu.cn (X.Y.); 20720231150072@stu.xmu.edu.cn (Y.L.); 2 Xiamen Key Laboratory of Electronic Ceramic Materials and Devices, College of Materials, Xiamen University, Xiamen 361005, China

**Keywords:** ultra-high temperature ceramics, single-source precursor, polymer-derived ceramics, phase evolution, thermodynamics and kinetics

## Abstract

In the present work, a series of single-source precursors were prepared via a one-pot synthesis strategy using transition metal chlorides, acetylacetone, and hydroquinone as raw materials. The molecular structure, cross-linking behavior, and polymer-to-ceramic transformation of the obtained precursors were systematically investigated by Fourier-transform infrared spectroscopy and thermogravimetric analysis. The phase composition, phase-transformation temperature, and grain size of the resulting ceramics were characterized by X-ray diffraction combined with Rietveld refinement. The resulting precursors exhibit good solubility in common organic solvents (e.g., ethanol, propanol, and acetone), rendering them suitable for fabricating ultra-high temperature ceramic matrix composites through polymer infiltration and the pyrolysis method. At 1400 °C, the ceramic yields of the TaC, HfC, ZrC, NbC, and TiC precursors were 62.45%, 57.53%, 48.55%, 45.53%, and 30.32%, respectively. After heat treatment at their respective phase-transformation temperatures, the resulting ceramics exhibited grain sizes of carbides in the range of approximately 80–100 nm. The mechanism governing the different phase-transformation temperatures (T) of the derived ceramics, which follow the order T_NbC_ < T_TaC_ < T_TiC_ < T_HfC_ < T_ZrC_, was elucidated through combined thermodynamic and kinetic analyses. This synthesis strategy was extended to the family of ultra-high temperature refractory metal carbides with melting points exceeding 3000 °C, demonstrating promising application potential for ultra-high temperature ceramic matrix composites.

## 1. Introduction

Driven by the rapid development of cutting-edge technologies such as hypersonic vehicles, reusable launch rockets, and advanced nuclear energy systems [1,2,3,4], extreme aerodynamic heating encountered during near-space flight and atmospheric re-entry has placed severe demands on the high-temperature stability, oxidation resistance, and thermomechanical integrity of thermal protection system (TPS) materials [5,6,7,8,9,10]. Metal alloys, carbon fiber-reinforced carbon matrix composites (C/C) and fiber-reinforced ceramic matrix composites (CMCs) are typical TPS materials. However, conventional metal alloys are precluded from high-temperature applications due to their relatively low melting points and the rapid degradation of mechanical strength at elevated temperatures [11,12,13,14]. Although C/C composites exhibit excellent high-temperature mechanical properties, they are highly vulnerable to oxidation in oxidizing atmospheres and therefore require external protective coatings [15,16]. Furthermore, CMCs such as silicon carbide-based composites (C/SiC) are subjected to active oxidation above 1650 °C, which impedes the formation of a protective oxide scale and thereby constrains their maximum service temperature [17,18]. Consequently, ultra-high temperature ceramics (UHTCs), characterized by melting points exceeding 3000 °C, excellent thermochemical stability, and superior oxidation resistance, have been regarded as promising candidates to address the limitations of current TPS materials for extreme environments [19,20,21,22,23,24]. Among these, the family of refractory metal carbides (MC, M = Hf, Ta, Nb, Zr, Ti) are distinguished by high hardness, high thermal conductivity, and excellent thermal shock resistance, rendering them particularly attractive for applications in thermal protection systems, rocket nozzles, and vehicle nose cones [17,18,25,26].

Currently, various approaches to preparing UHTC powders have been developed, including carbothermal reduction [27], self-propagating high-temperature synthesis [28], and plasma sintering [29]. However, these conventional methods show numerous limitations, including elevated synthesis temperatures, prolonged processing cycles, limited product purity, excessively large grain sizes, and non-uniform phase distribution. Although the sol–gel method [30,31] can reduce the synthesis temperature, it remains constrained by intrinsic limitations, including inadequate precursor stability, prolonged process cycles, and complex protocols, thereby restricting its scalability. In contrast, the polymer-derived ceramic (PDC) method has emerged as an advanced and efficient approach for the preparation of UHTC nanopowders and composites [32,33,34,35,36], owing to its unique advantages, including precise compositional and structural design at the molecular level, low-temperature shaping, and the facilitation of complex-shaped ceramic materials.

In recent years, considerable progress has been achieved in the development of both oxygen-free and oxygen-containing precursors for carbide systems. Regarding the oxygen-free route, Wang et al. [37] prepared metallocene-based precursors containing Zr, Hf, or Zr/Hf through the alkylation reaction between cyclopentadienyl ligands and methylene, which were subsequently converted into HfC/ZrC ceramics at 1400 °C, with ceramic yields of 57.50–66.34%. However, these precursors are highly sensitive to moisture and oxygen, being non-melting and insoluble, thereby limiting their processability and restricting their applicability in the polymer infiltration and pyrolysis (PIP) process for fabricating UHTC-modified CMCs. In contrast, oxygen-containing precursors have attracted considerable attention due to their good air stability, controllable and facile preparation conditions, and favorable solubility. Tao et al. [38] synthesized a ZrC precursor through a one-pot reaction of zirconium oxychloride (ZrOCl_2_·8H_2_O) and 2-hydroxybenzyl alcohol; however, the resulting ZrC ceramics exhibited relatively large grain sizes of 100–300 nm upon heat treatment. Jiang et al. [39] synthesized a HfC precursor using hafnium tetrachloride, sucrose and polyvinylpyrrolidone as raw materials. After carbothermal reduction at 1600 °C, the precursor was converted into a pure HfC phase with an average particle size of approximately 73 nm; nevertheless, the ceramic yield was only 46.3%. In oxygen-containing preparation methods, existing UHTC carbide precursors are still hindered by several critical drawbacks, as follows: (i) excessively large grain sizes in the resulting ceramic phase, (ii) elevated temperatures required for the oxide-to-carbide transformation, and (iii) the lack of extension of the application scope to the entire family of ultra-high-temperature refractory metal carbides. These limitations collectively preclude their use as ideal impregnating agents for PIP-based fabrication of CMCs. Very recently, we reported a series of novel soluble oxygen-containing precursors for HfC and ZrC, employing metal chlorides (e.g., HfCl_4_, ZrCl_4_) as metal sources and methacrylic anhydride (MA) and hydroquinone (DHB) as carbon sources [26,40]. The incorporation of C=C double bonds further enhanced the ceramic yield, with the HfC precursor achieving up to 58.98% at 1500 °C. Our previous efforts were confined to single-carbide systems (HfC and ZrC); nevertheless, a comprehensive investigation of the UHTC carbide family has yet to be conducted, and the kinetic and thermodynamic mechanisms governing the ceramic phase-transformation temperatures remain elusive.

Building upon our previous work [26,40], the present work presents a universal synthesis strategy for precursors applicable to the family of ultra-high temperature refractory metal carbides with melting points exceeding 3000 °C, including HfC, TaC, NbC, ZrC and TiC. A series of single-source precursors, denoted as MDA (M = Hf, Ta, Nb, Zr, Ti), were synthesized via a one-pot synthesis strategy using metal chlorides (MCl_x_), acetylacetone (Acac), and DHB as raw materials. The acronym MDA is derived from the key components used in its synthesis, where M represents metal chlorides (MCl_x_), D represents 1,4-dihydroxybenzene, and A represents acetylacetone. Subsequent cross-linking, pyrolysis, and annealing afforded the corresponding carbide ceramics (MC, M = Hf, Ta, Nb, Zr, Ti). In the present work, the MDA precursors were characterized, and the evolution of molecular structure, phase composition, and grain size during the polymer-to-ceramic transformation was analyzed and discussed. Additionally, the mechanism underlying the oxide-to-carbide phase-transformation temperatures was elucidated through combined thermodynamic and kinetic analyses.

## 2. Results and Discussion

### 2.1. Characterization of the Single-Source Precursors

**Figure 1:**
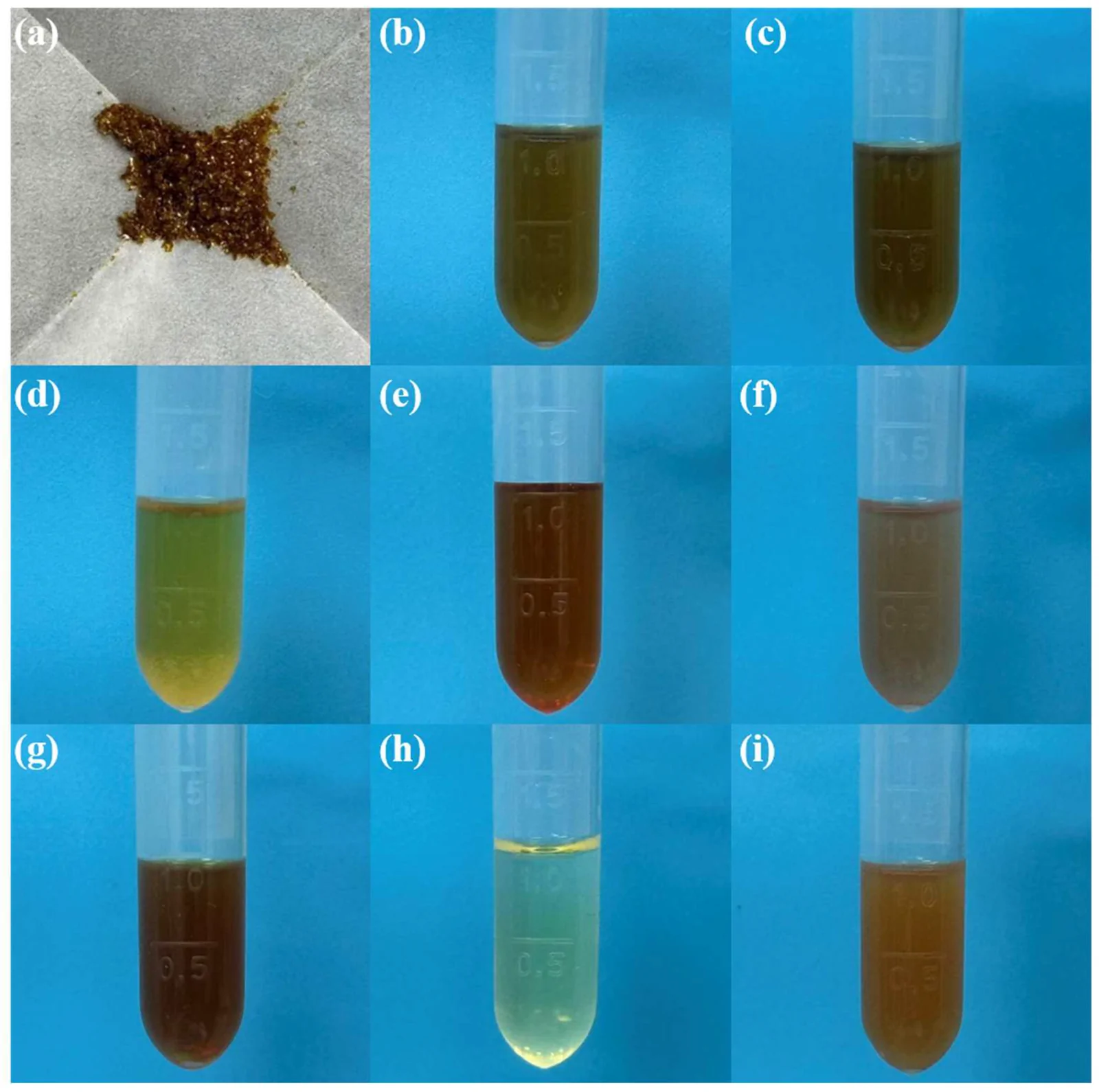
(**a**) Dried TaDA powders and TaDA dissolved in (**b**) ethanol, (**c**) propanol, (**d**) acetone, and (**e**) DMF, and (**f**) HfDA, (**g**) NbDA, (**h**) ZrDA, and (**i**) TiDA dissolved in ethanol.

To evaluate the solubility of the as-synthesized single-source precursors (MDA, M = Hf, Ta, Nb, Zr, Ti), the precursor powders prior to cross-linking were subjected to solubility tests in common organic solvents. The TaDA appears as a brownish-yellow powder (Figure 1a). For the solubility assessment, 30 mg of TaDA powder was dissolved in 1 mL each of ethanol, propanol, acetone, and N, N-dimethylformamide (DMF), respectively. As shown in Figure 1b–e, TaDA was found to be readily soluble in all the tested solvents. Similarly, 30 mg of each of the HfDA, NbDA, ZrDA, and TiDA precursors was dissolved in 1 mL of anhydrous ethanol, and all were found to exhibit good solubility (Figure 1f–i).

These results demonstrate that the MDA precursors are readily soluble in polar solvents such as ethanol, rendering them suitable as impregnating agents for the PIP process to fabricate CMCs. Additionally, the compatibility of these precursors with ethanol, as a low-toxicity, widely available, and cost-effective solvent, highlights their processability and sustainability, thereby promoting the practical application of UHTCs in thermal protection systems.

**Figure 2:**
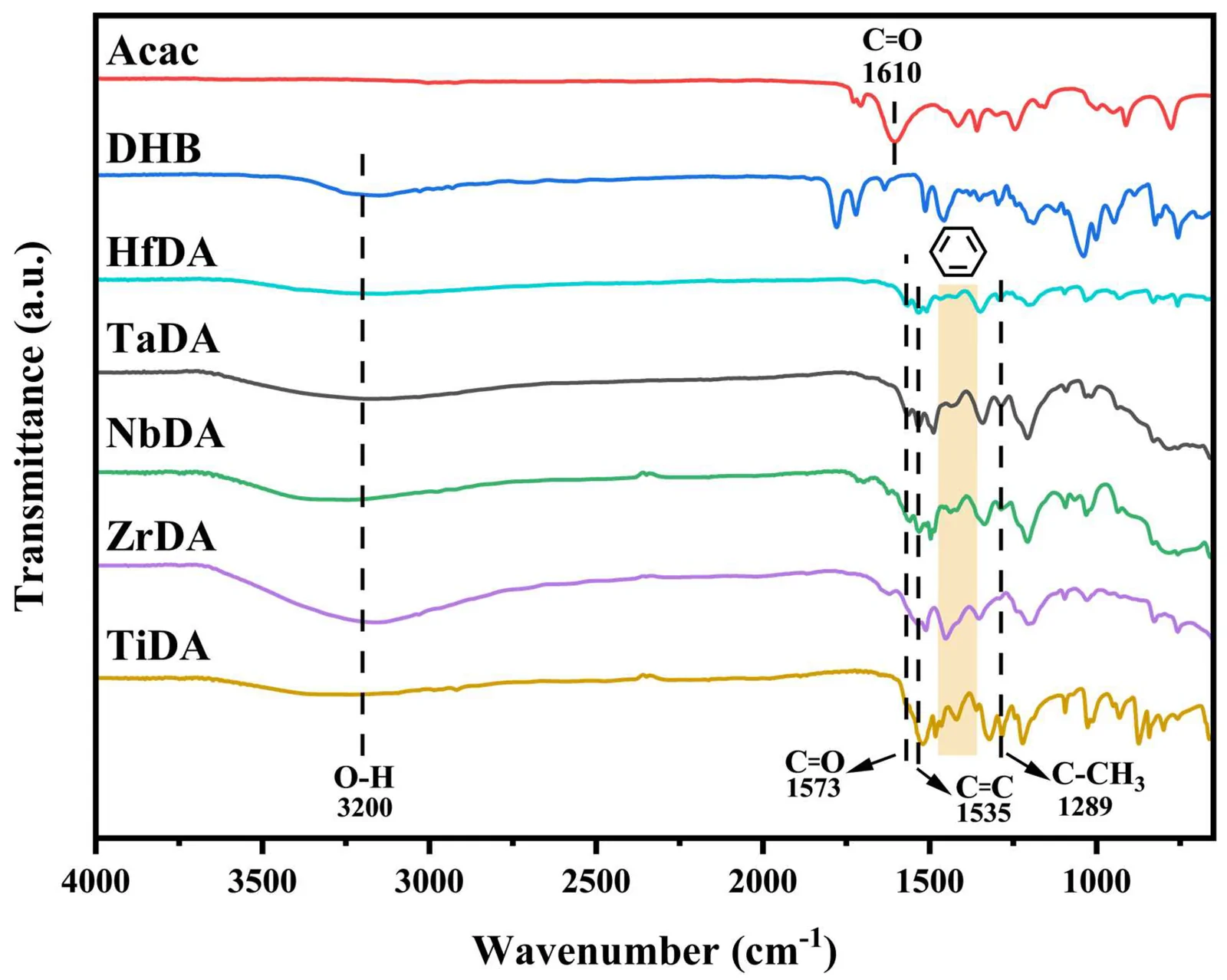
FT-IR spectra of the MDA precursors.

To further investigate the structural evolution of the MDA precursors during the synthesis process, FT-IR analysis was performed, and the results are shown in Figure 2.

**Figure 3:**
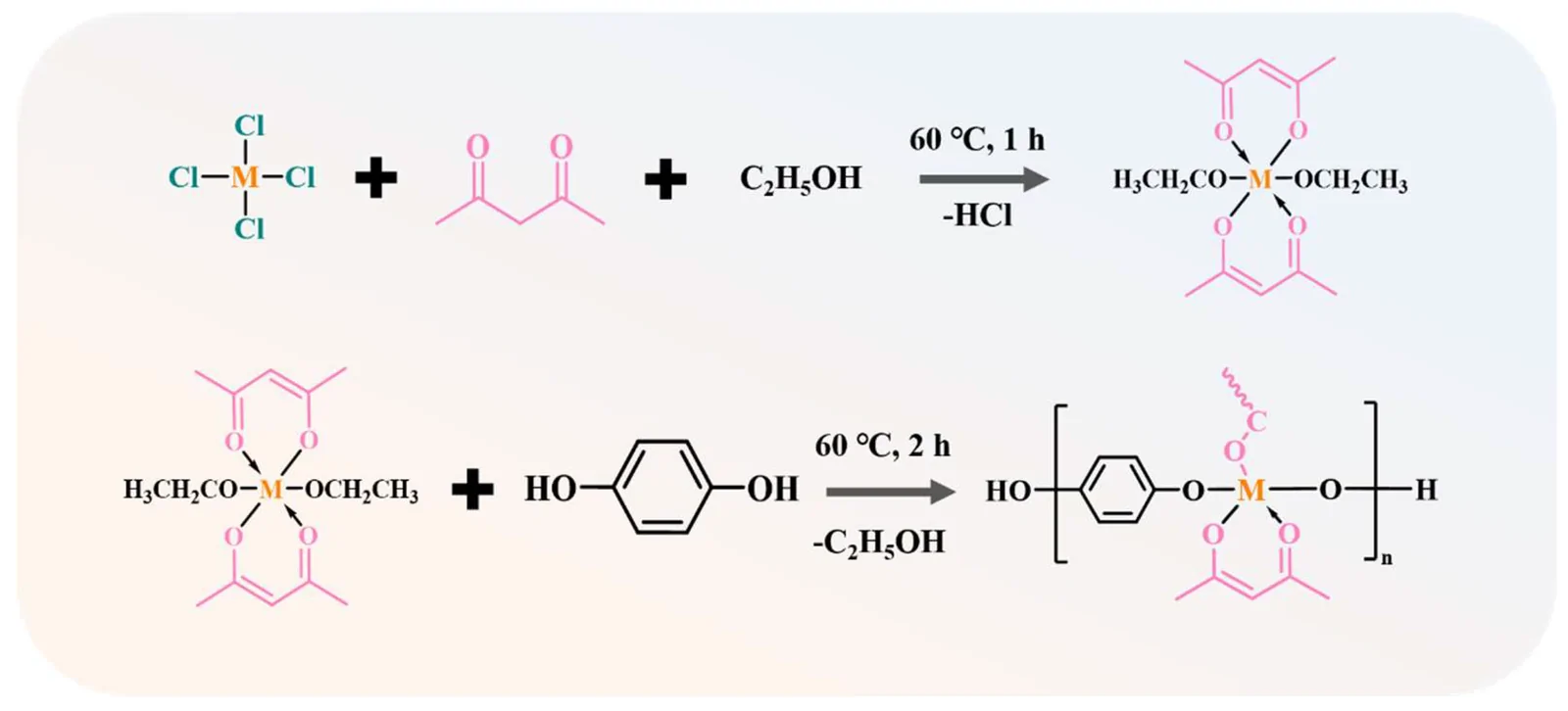
Schematic diagram of the synthesis mechanism of the precursors.

The FT-IR spectra of all MDA precursors exhibit two characteristic absorption peaks at approximately 1573 cm^−1^ and 1535 cm^−1^, attributed to the coupled stretching vibrations of C=O and C=C in the coordinated Acac, indicative of metal-acetylacetonate chelate ring formation. Relative to the C=O stretching vibration of free Acac at 1610 cm^−1^, this vibration in all precursors shows a significant redshift, indicative of coordination between the carbonyl oxygen atoms of Acac and the respective transition metal ions (Hf^4+^, Ta^5+^, Nb^5+^, Zr^4+^, Ti^4+^) [41]. Additionally, all spectra reveal a benzene ring skeletal vibration peak at approximately 1360 cm^−1^, confirming the incorporation of DHB into the molecular architecture of the precursors. These characteristic peaks demonstrate that the molecular design strategy employing Acac as a chelating ligand and DHB as an aromatic bridging unit is universally applicable to the synthesis of UHTC-carbide precursors. The proposed synthesis mechanism is illustrated in Figure 3.

### 2.2. Cross-Linking of the Single-Source Precursors

**Figure 4:**
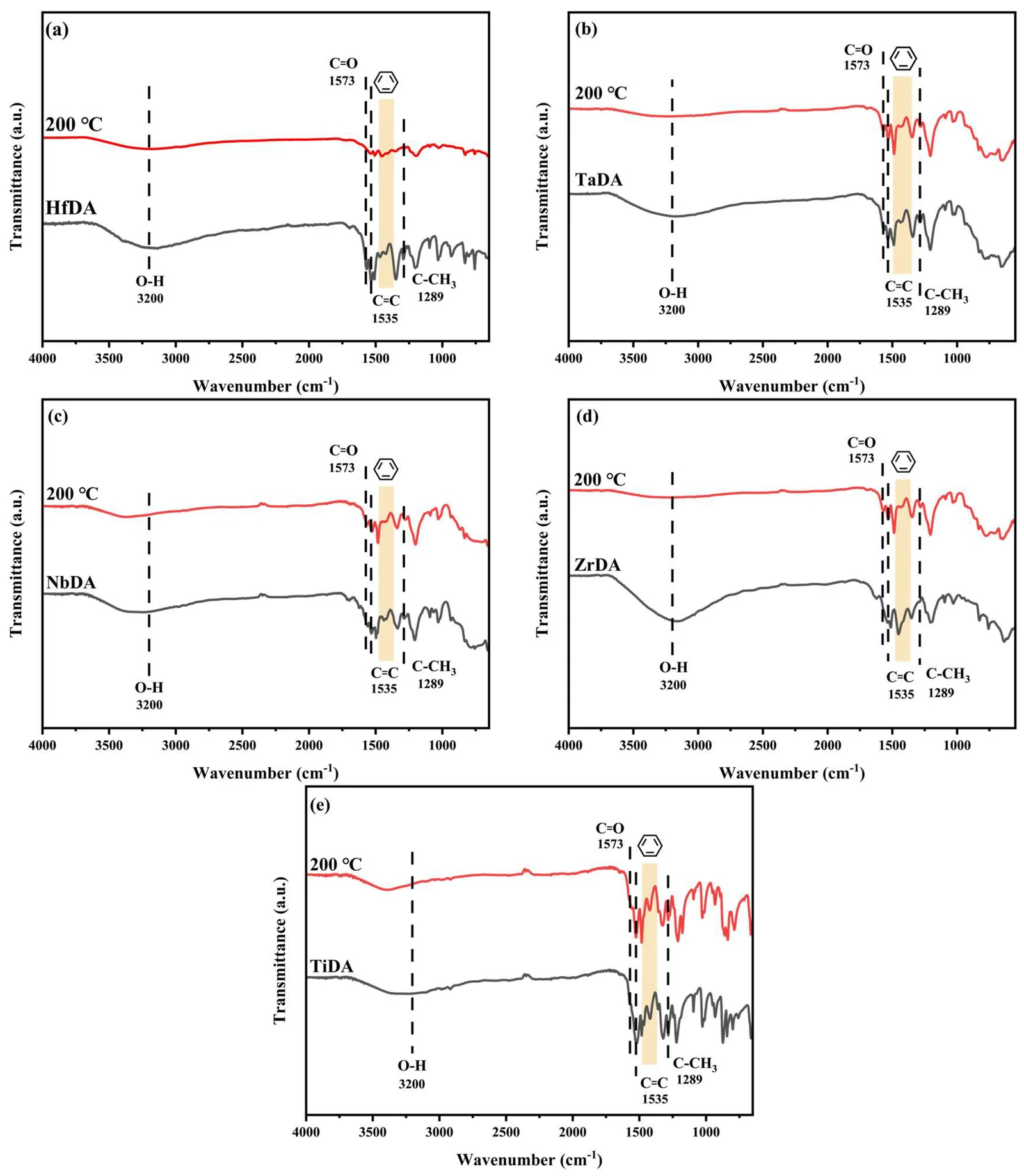
FT-IR spectra of the MDA precursors (**a**) HfDA, (**b**) TaDA, (**c**) NbDA, (**d**) ZrDA and (**e**) TiDA before and after cross-linking at 200 °C.

**Figure 5:**
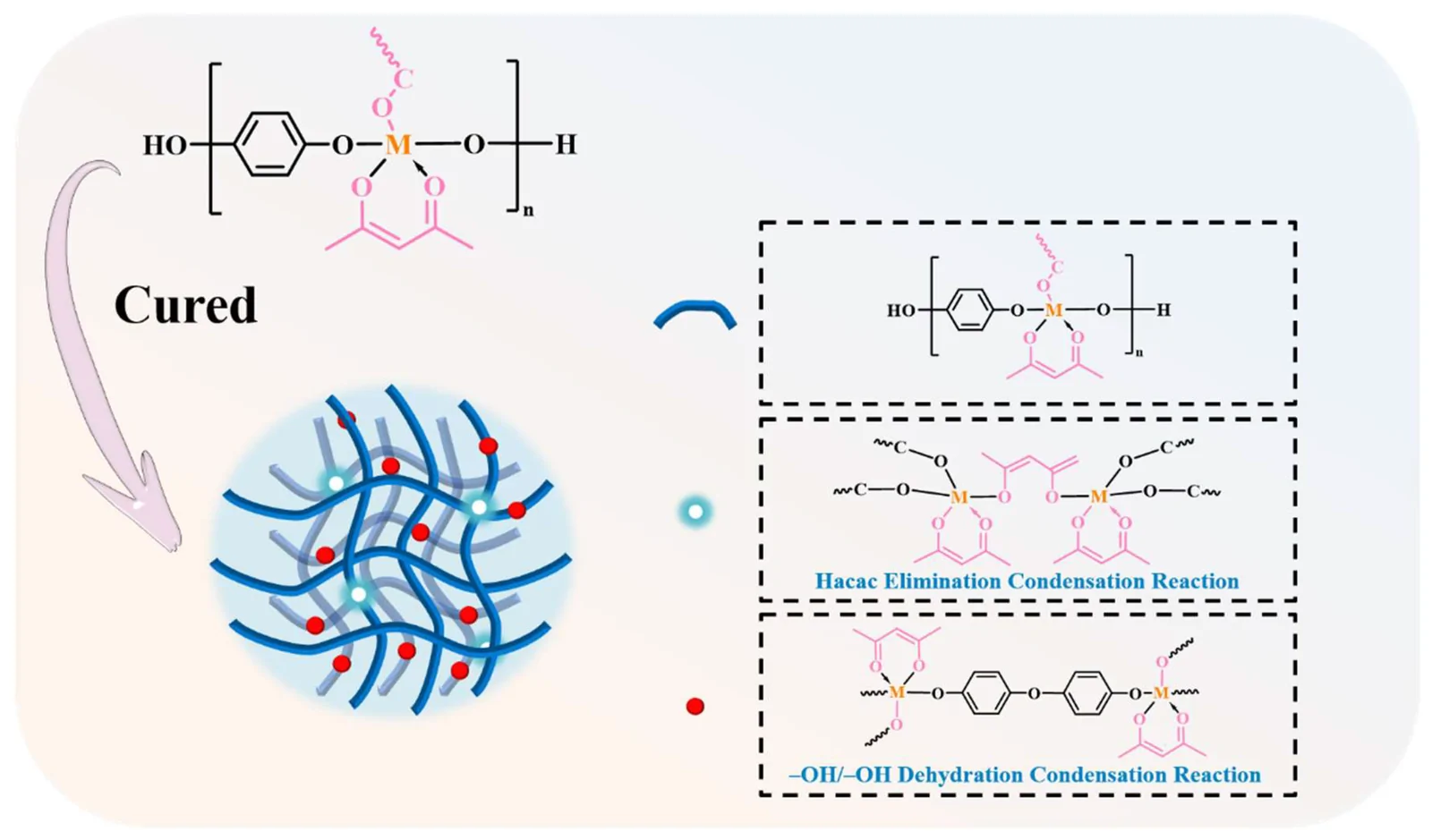
Schematic illustration of the cross-linking mechanism of the MDA (M = Hf, Ta, Nb, Zr, Ti) precursors at 200 °C.

In general, cross-linking is a critical step in the PDC route, as it converts the precursors into an insoluble and non-fusible network that retains the desired shape and enhances the ceramic yield during subsequent pyrolysis [42]. Figure 4 compares the FT-IR spectra of the series of MDA precursors before and after cross-linking. After cross-linking at 200 °C, the characteristic peaks assigned to C=O and C=C stretching vibrations of the Acac ligand were retained but with reduced intensity, which is attributable to condensation reactions during the cross-linking process. Typically, (i) the six-membered ring structure of certain Acac ligands was disrupted with the loss of a hydrogen atom, thereby leading to interconnection of metal centers through Acac bridges; (ii) another fraction of the Acac ligands was detached from the metal centers, yielding Hacac as a by-product, consistent with the previous literature [41,43,44]. Additionally, the broad absorption peak at approximately 3200 cm^−1^ associated with the ─OH groups of DHB was markedly attenuated after cross-linking, indicating the occurrence of a dehydration condensation reaction (─OH/─OH). These results suggest that cross-linking proceeds primarily through two pathways: (i) ─OH/─OH dehydration condensation and (ii) Hacac elimination from the Acac ligand. The proposed cross-linking mechanism is illustrated in Figure 5.

### 2.3. Polymer-to-Ceramic Transformation

**Figure 6:**
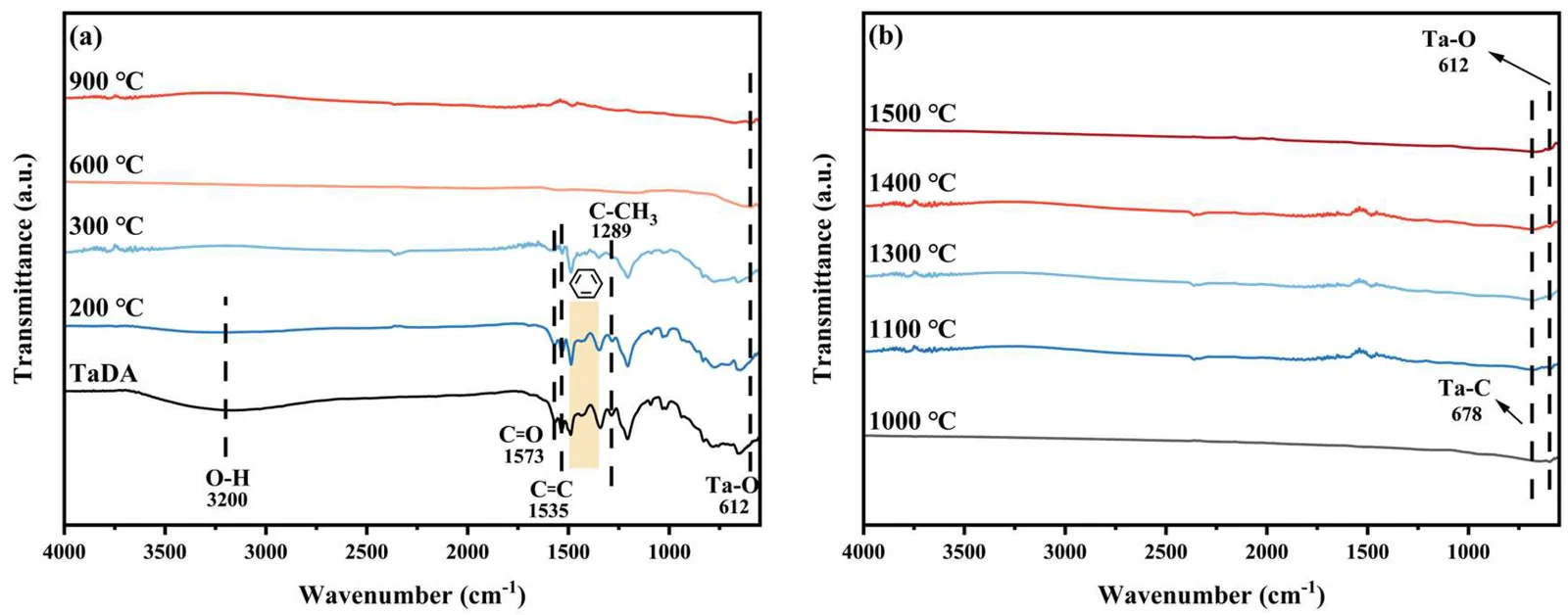
FT-IR spectra of the TaDA precursor after heat treatment at different temperatures: (**a**) 200 °C–900 °C and (**b**) 1000 °C–1500 °C.

To investigate the structural evolution during the polymer-to-ceramic transformation, FT-IR analysis was performed on TaDA as a representative example after heat treatment at various temperatures (Figure 6). As shown in Figure 6a, at the pyrolysis temperature of 300 °C, the C=O absorption peak of the Acac ligand at 1573 cm^−1^ disappears, which is attributable to the disruption of its six-membered chelate ring. At the pyrolysis temperature of 600 °C, the absorption peaks corresponding to organic groups almost vanish, leaving solely the characteristic peak of Ta-O bonds at 612 cm^−1^. This indicates that the precursor has essentially completed its transformation to an inorganic state at this temperature. In the present work, this stage is defined as the pyrolysis step, during which the organic precursor is converted into an inorganic framework. Subsequent heat treatment at higher temperatures is referred to as the annealing step, during which the ceramic powders undergo phase transformation at elevated temperatures.

Figure 6b shows that when the temperature is raised to the annealing temperature of 1300 °C, the Ta-O bond absorption peak is reduced and broadened, and a new absorption peak assigned to the Ta-C bond emerges at 678 cm^−1^ [25], indicating the initiation of the carbothermal reduction of Ta_2_O_5_ to form TaC. At the annealing temperature of 1400 °C, the Ta-O absorption peaks are nearly undetectable, and only the Ta-C absorption peaks are observed. This confirms the complete conversion of Ta_2_O_5_ to TaC. The FT-IR results demonstrate that the inorganic transformation of the precursor is almost complete at 600 °C, with Ta_2_O_5_ formation occurring before its conversion to TaC at 1400 °C, which is consistent with the subsequent XRD characterization.

**Figure 7:**
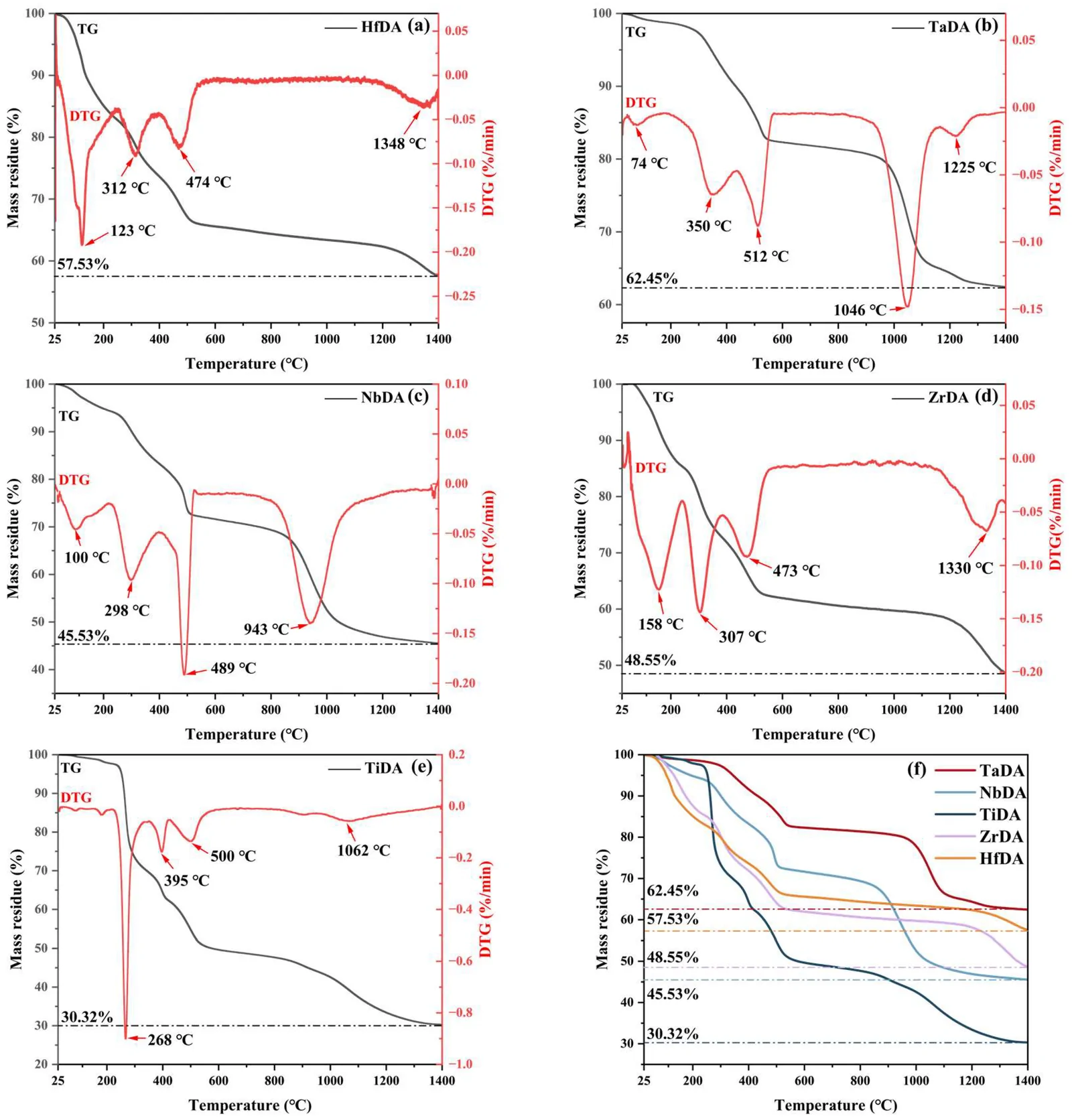
(**a**–**e**) TG and DTG curves of the MDA precursors after cross-linking at 200 °C, and (**f**) TG curves of the cross-linked MDA precursors.

To investigate the polymer-to-ceramic transformation of the MDA (M = Hf, Ta, Nb, Zr, Ti) precursors, thermogravimetric analysis (TGA) and first-order derivative of TG (DTG) were conducted on the cross-linked precursors under an Ar atmosphere from room temperature to 1400 °C, with the results shown in Figure 7. In general, the thermal decomposition behaviors of all five precursors were categorizable into four main stages: (i) the first stage (RT-350 °C) is attributed to the volatilization of residual solvents and low-molecular-weight oligomers, with a mass loss of ca. 10–30 wt%; notably, the TaDA precursor exhibits the lowest mass loss (∼10 wt%) in this range; (ii) the second stage (350–550 °C) is associated with a mass loss of ca. 8–21 wt%, due to the cleavage and decomposition of organic groups (e.g., Acac ligands and DHB bridging units), with the release of gaseous by-products [45]; the TaDA precursor shows the minimum mass loss (∼8 wt%) in this stage; (iii) the third stage (550 °C–1000 °C) displays a weight loss of only ca. 3–5 wt.% for all precursors, indicating that the transformation from the polymer network to an inorganic skeleton is almost completed, as no significant weight loss occurs in this stage; and (iv) the fourth stage (>1000 °C) again shows a weight loss of about 5–17 wt.%. Initially, the oxide grains grow with increasing temperature, but no significant mass loss is observed until the annealing temperature approaches the predicted carbothermal reduction temperature, at which point a considerable weight loss occurs. As reported in the literature [46,47,48,49,50], this weight loss is attributed to the carbothermal reduction reaction between the metal oxides (MO_x_) and free carbon to form the target MC phase, mainly releasing CO gas, as described by Equation (1) [51]. The completion of this transformation is further confirmed by the subsequent XRD analysis.

M_x_O_y_ + (x + y) C→xMC + yCO↑ (M = Hf, Ta, Nb, Zr, Ti) (1)

For comparison, Figure 7f summarizes the ceramic yields of the five precursors at 1000 °C and 1400 °C. At 1000 °C, the yields are 77.82%, 63.40%, 59.73%, 52.55%, and 42.56% for TaDA, HfDA, ZrDA, NbDA, and TiDA, respectively; at 1400 °C, the corresponding values are 62.45%, 57.53%, 48.55%, 45.53%, and 30.32%. The ceramic yields follow the order TaDA > HfDA > ZrDA > NbDA > TiDA. Among the five cross-linked precursors, TaDA possesses the highest ceramic yield at both temperatures, while TiDA exhibits the lowest. To further elucidate the differences in ceramic yield among the various precursors, we have excluded the influence of the different M:Acac ratios by dividing the five systems into two groups based on metal valence (pentavalent and tetravalent). For the pentavalent metal precursors (M:Acac = 1:4.25), the relative atomic mass of Ta (180.95) is larger than that of Nb (92.91), and the ceramic yield of TaDA (62.45%) is correspondingly higher than that of NbDA (45.53%). For the tetravalent metal precursors (M:Acac = 1:3), the relative atomic masses decrease in the order Hf (178.49) > Zr (91.22) > Ti (47.87), and the ceramic yields exhibit the same trend: HfDA (57.53%) > ZrDA (48.55%) > TiDA (30.32%). Therefore, it is reasonable to infer that, for metals with the same valence state, a higher relative atomic mass of the metal leads to a higher metal content in the precursor, and consequently a higher ceramic yield. In comparison with the literature, our precursors exhibit competitive ceramic yields for TiC, HfC, and TaC. The TiDA yield (30.32% at 1400 °C) exceeds that of a reported TiC precursor (approximately 30% at 1200 °C) [52]; the HfDA yield (57.53% at 1400 °C) is higher than that of a reported HfC precursor (approximately 46.3% at 1600 °C) [39]; and the TaDA yield (62.45% at 1400 °C) surpasses the reported value of 54.02% [53], demonstrating competitive advantages in ceramic yield over the reported precursors.

### 2.4. Phase Composition of Ceramics

**Figure 8:**
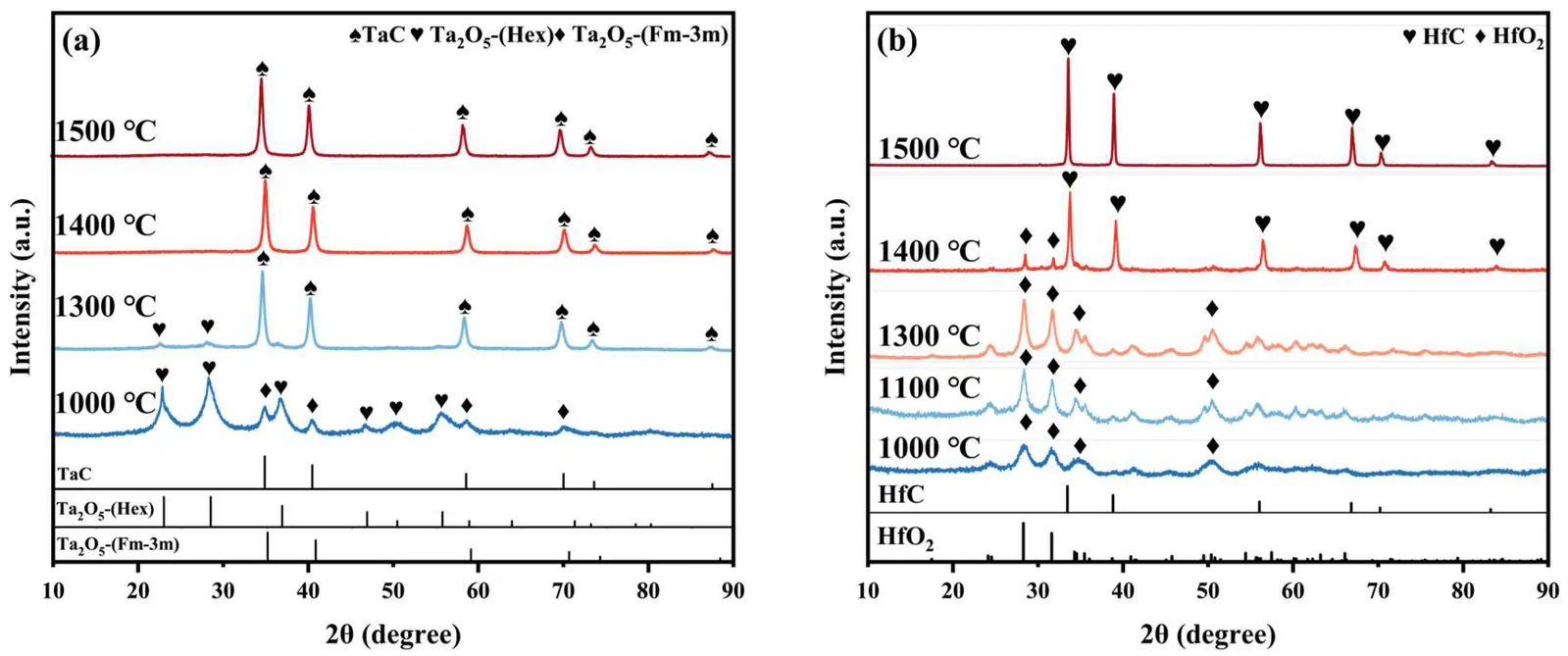

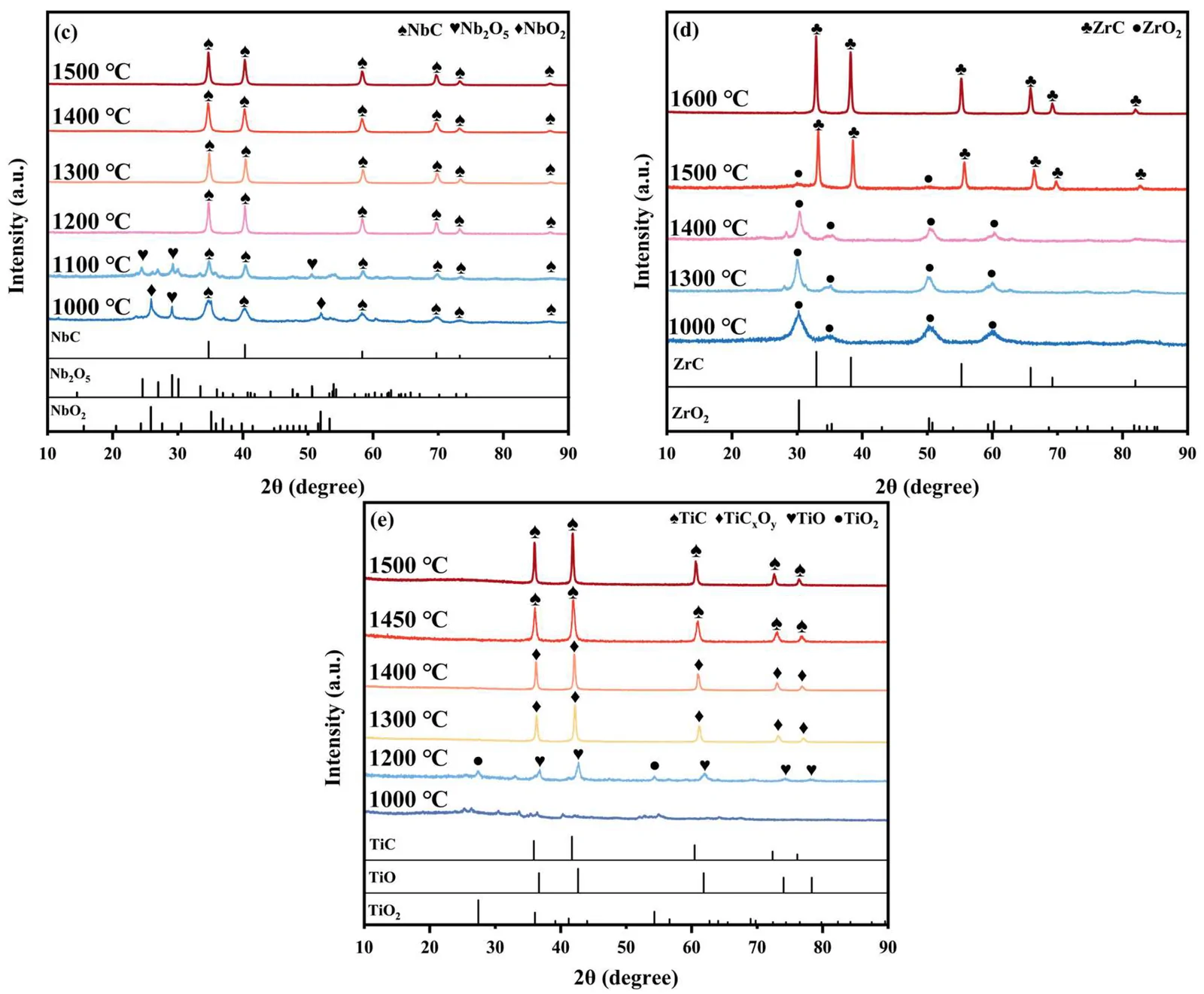
XRD patterns of the MDA-derived ceramics after heat treatment at different temperatures: (**a**) TaDA, (**b**) HfDA, (**c**) NbDA, (**d**) ZrDA, and (**e**) TiDA.

To investigate the phase evolution of the MDA-derived ceramics during the pyrolysis, XRD analysis was performed on samples annealed at various temperatures, and the results are shown in Figure 8. Figure 8a presents the XRD patterns of the TaDA-derived ceramics annealed at various temperatures. Upon heat treatment at 1000 °C, both hexagonal Ta_2_O_5_ (JCPDS No. 18-1304) and face-centered cubic Ta_2_O_5_ (JCPDS No. 78-0724) are detected, with the broad diffraction peaks indicative of low crystallinity. When the temperature is elevated to 1300 °C, the intensity of Ta_2_O_5_ diffraction peaks reduces, while those of TaC (JCPDS No. 35-0801) appear. The TaC diffraction peaks at 2θ = 34.855°, 40.460°, 58.559°, 70.003°, and 87.501° correspond to the (111), (200), (220), (311), and (400) crystal planes, respectively, consistent with (JCPDS No. 35-0801), attributable to the onset of carbothermal reduction and partial conversion of Ta_2_O_5_ to TaC. At 1400 °C, the Ta_2_O_5_ diffraction peaks are no longer detected, and the TaC phase is exclusively present, confirming the near-completion of the oxide-to-carbide transformation. At 1500 °C, the TaC diffraction peaks become markedly sharper with increased intensity, signifying enhanced crystallinity.

**Figure 9:**
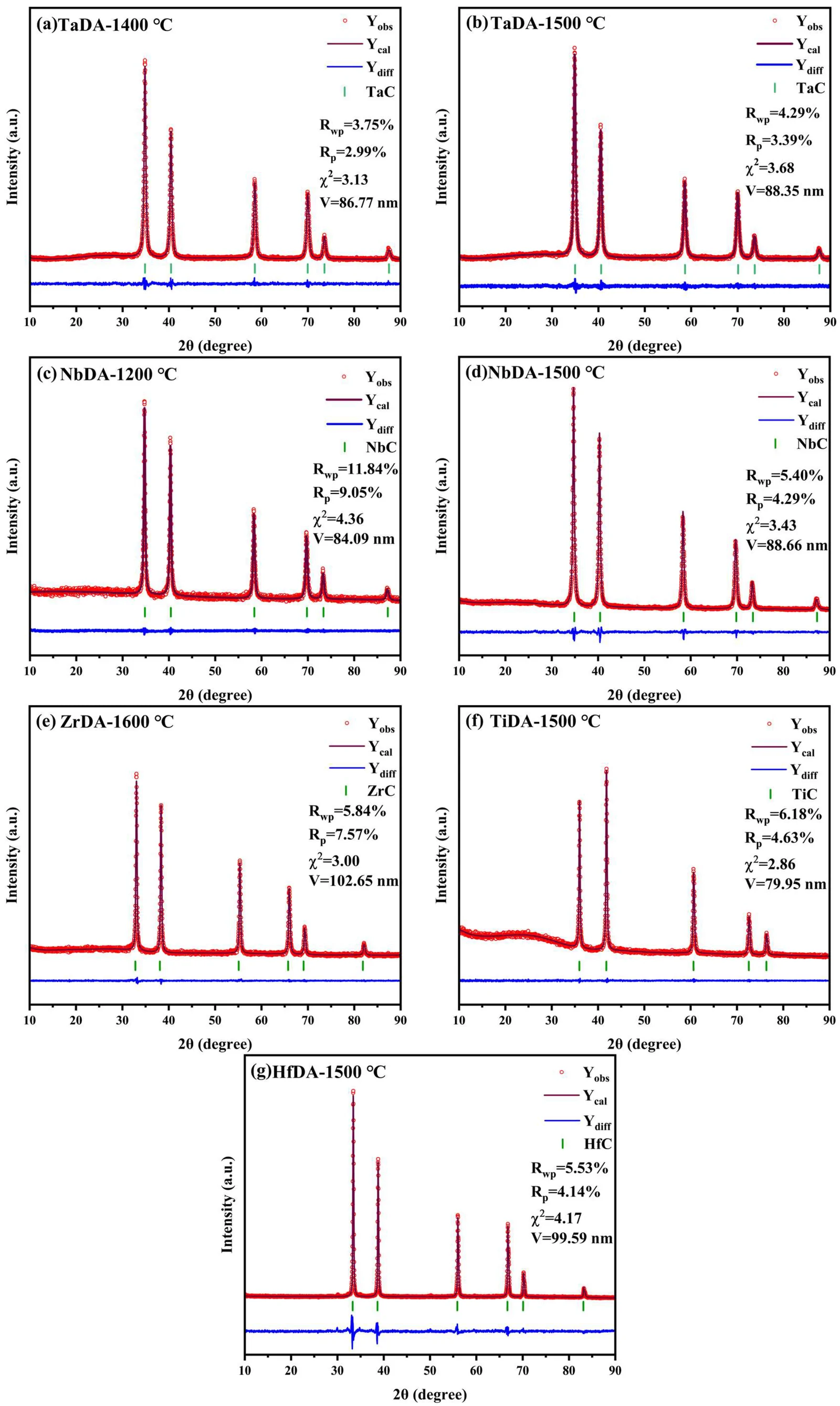
Rietveld refinement results of the X-ray diffraction patterns of the MDA precursors-derived ceramics after heat treatment at different temperatures: (**a**) TaDA-1400 °C, (**b**) TaDA-1500 °C, (**c**) NbDA-1200 °C, (**d**) NbDA-1500 °C, (**e**) ZrDA-1600 °C, (**f**) TiDA-1500 °C, and (**g**) HfDA-1500 °C.

Similarly, the HfDA, NbDA, ZrDA, and TiDA precursors undergo comparable phase evolution upon high-temperature annealing (Figure 9b–e). Figure 8b shows that the HfDA precursor is fully converted into HfC (JCPDS No. 73-0475) at 1500 °C, displaying diffraction peaks at 2θ = 33.414°, 38.774°, 55.996°, 66.799°, 70.192°, and 83.195°, which correspond to the (111), (200), (220), (311), (222), and (400) planes, respectively. As shown in Figure 8c, the NbDA precursor is completely transformed into NbC (JCPDS No. 38-1364) upon annealing at 1200 °C. In addition, the resulting diffraction peaks at 2θ = 34.730°, 40.316°, 58.337°, 69.718°, 73.306°, and 87.139° are indexed to the (111), (200), (220), (311), (222), and (400) planes, respectively. The Figure 8d reveals that the ZrDA precursor is thoroughly converted into ZrC (JCPDS No. 35-0784) at 1600 °C, exhibiting diffraction peaks at 2θ = 33.040°, 38.337°, 55.323°, 65.967°, 69.304°, and 82.049° that are assigned to the (111), (200), (220), (311), (222), and (400) planes, respectively. The Figure 8e indicates that the TiDA precursor is completely converted into TiC (JCPDS No. 32-1383) by high-temperature heat treatment at 1450 °C, as confirmed by diffraction peaks at 2θ = 35.906°, 41.710°, 60.448°, 72.369°, and 76.139° corresponding to the (111), (200), (220), (311), and (222) planes, respectively. All five precursors afford the target carbides through the identical synthesis and pyrolysis route; nevertheless, the oxide-to-carbide phase-transformation temperatures varies markedly, following the order:

T_ZrC_ > T_HfC_ > T_TiC_ > T_TaC_ > T_NbC_

In the annealing temperature range of 1500–1600 °C, all obtained ceramics were completely transformed into the carbide MC phase. Rietveld refinements of the XRD patterns were performed using the GASA 2 software (Figure 9), with peak shapes fitted using the pseudo-Voigt function. The resulting R_wp_ values are all below 12%, indicating good agreement between the refined and experimental data. The samples are designated as MDA-T, where M represents the metal element and T denotes the annealing temperature (e.g., TaDA-1500 °C refers to TaDA-derived TaC annealed at 1500 °C). The refined grain sizes of the resulting carbides are 88.35 nm for TaC (TaDA-1500 °C), 88.66 nm for NbC (NbDA-1500 °C), 102.65 nm for ZrC (ZrDA-1600 °C), 79.95 nm for TiC (TiDA-1500 °C), and 99.59 nm for HfC (HfDA-1500 °C), falling in the range of approximately 80–100 nm. Notably, increasing the heat treatment temperature from 1400 to 1500 °C results in a slight grain growth of TaC (from 86.77 to 88.35 nm), while NbC shows a similar increase from 84.09 nm at 1200 °C to 88.66 nm at 1500 °C. These results demonstrate that the ceramics derived from these precursors exhibit excellent resistance to grain coarsening at elevated temperatures, which is beneficial for maintaining their high-temperature mechanical properties [54,55,56,57].

### 2.5. Thermodynamic and Kinetic Analysis

To investigate the variations in oxide-to-carbide phase-transformation temperatures among the different precursors, thermodynamic simulations were conducted using HSC Chemistry 6.0 software. The XRD analysis reveals that the oxide-to-carbide transformation temperatures follow the order T_ZrC_ (1600 °C) > T_HfC_ (1500 °C) > T_TiC_ (1450 °C) > T_TaC_ (1400 °C) > T_NbC_ (1200 °C), which is in good agreement with the thermodynamically calculated sequence. The observed temperature sequence may be attributed to the complex interplay between thermodynamic driving forces and kinetic barriers inherent to the carbothermal reduction process in each system.

**Figure 10:**
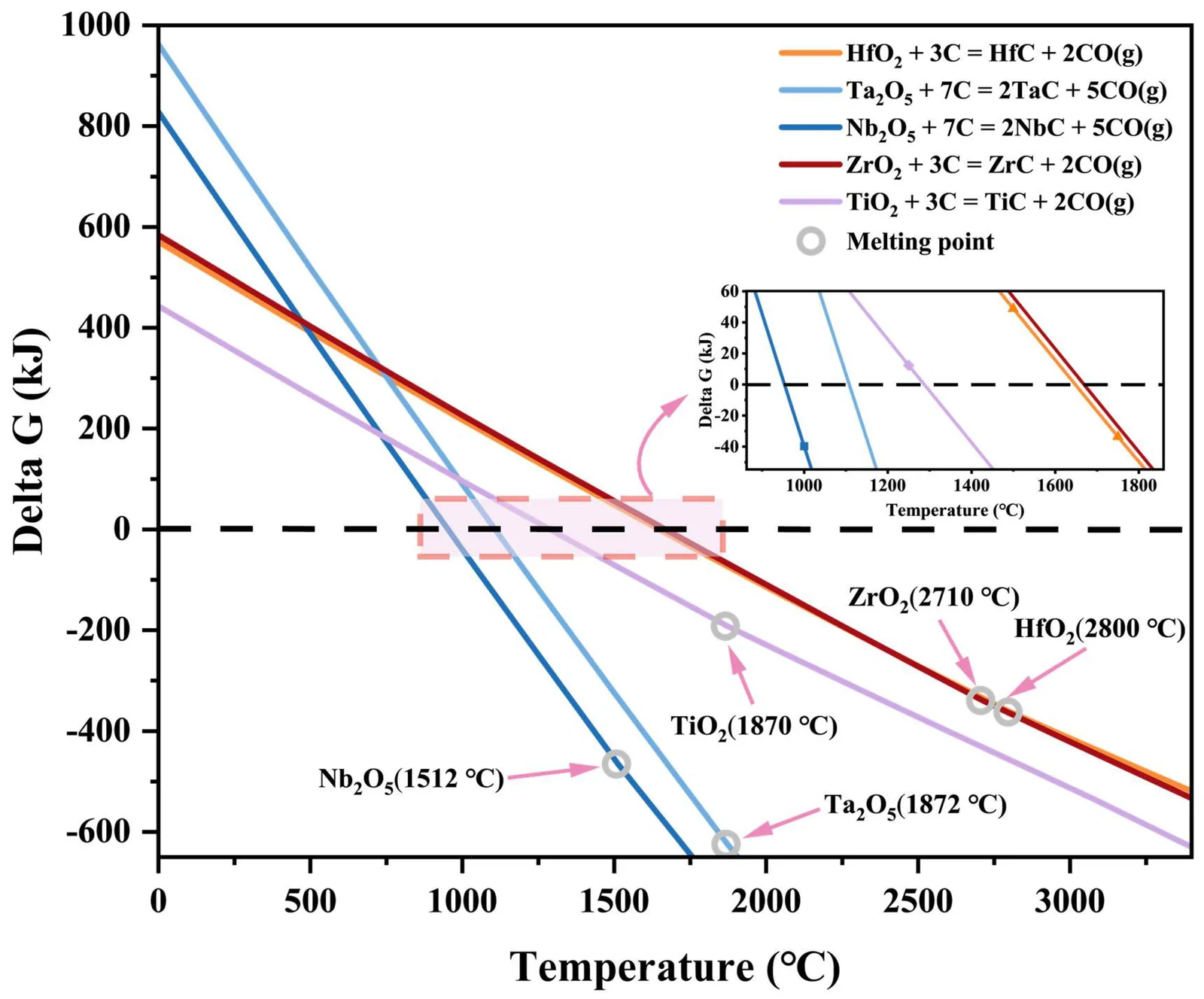
Gibbs free energy changes during the carbothermal reduction process calculated using HSC Chemistry 6.0 software.

From a thermodynamic perspective, the driving force for the carbothermal reduction reaction (M_x_O_y_ + (x + y)C = Xmc + yCO↑) is dictated by the Gibbs free energy change (ΔG) [58,59,60]. Generally, a more negative ΔG value corresponds to a greater thermodynamic driving force for the reaction. As illustrated in Figure 10, ZrO_2_ exhibits the least thermodynamically favored ΔG for carbothermal reduction among the oxides investigated, reflecting its greatest thermodynamic stability. Consequently, a higher temperature is required to provide sufficient driving force for the reduction of ZrO_2_ to ZrC. Conversely, Nb_2_O_5_ exhibits the most thermodynamically favored ΔG for carbothermal reduction, corresponding to the strongest driving force and thus enabling its conversion to NbC at substantially lower temperatures.

From a kinetic perspective, the lattice stability of metal oxides directly governs the ease of atomic diffusion during carbothermal reduction [61]. The melting point (T_m_) of the oxide provides a macroscopic measure of its lattice strength and metal–oxygen bond energy. A higher T_m_ is indicative of stronger metal–oxygen bonding and, consequently, greater resistance to lattice disruption, thereby necessitating a higher activation energy (E_a_) for carbon diffusion and reaction [62,63,64,65]. The relationship between the atomic diffusion coefficient (D) and temperature (T) is given by the Arrhenius equation [66] (Equation (2)):

(2) $$D=D_{0}exp(\frac{-E_{a}}{RT})$$

This equation indicates that a higher E_a_ corresponds to a lower diffusion coefficient D at a given temperature, thereby impeding atomic migration. As shown in Figure 10, the melting points of ZrO_2_ (~2710 °C) and HfO_2_ (~2800 °C) are significantly higher than those of TiO_2_ (~1870 °C) and Ta_2_O_5_ (~1872 °C), whereas Nb_2_O_5_ (~1512 °C) exhibits the lowest melting point [20,67]. Accordingly, the strong lattice stability and high diffusion barriers of ZrO_2_ and HfO_2_ necessitate carbothermal reduction temperatures of 1600 °C and 1500 °C, respectively. In contrast, the lower melting point and reduced lattice energy of Nb_2_O_5_ enable its transformation from oxide to carbide at 1200 °C. The kinetic behaviors of TiC and TaC are intermediate between these two extremes, consistent with the experimentally observed phase-transformation temperature sequence:

T_ZrC_ > T_HfC_ > T_TiC_ > T_TaC_ > T_NbC_

Compared with the previously reported literature, the MDA precursors afford complete conversion to the MC phase at markedly lower temperatures (e.g., NbC at 1200 °C, TaC at 1400 °C, and TiC at 1450 °C), which suggests potential advantages of this approach over conventional carbothermal reduction [68,69,70]. These low phase-transformation temperatures make the precursors particularly attractive for fabricating CMCs through the PIP approach, by avoiding the severe mechanical degradation of carbon fibers typically encountered at temperatures exceeding 1500 °C [71,72]. As a result, the observed sequence of phase-transformation temperatures is attributed to the combined effects of thermodynamic driving forces and kinetic diffusion barriers associated with the respective metal oxides.

## 3. Experimental Methods

### 3.1. Materials

HfCl_4_ (purity: 99.5%), TaCl_5_ (purity: 99.9%), NbCl_5_ (purity: 99.9%), ZrCl_4_ (purity: 98.0%), and TiCl_4_ (purity: 99.0%) were purchased from Shanghai Macklin Biochemical Co., Ltd. (Shanghai, China). Anhydrous ethanol (AR) was supplied by Sinopharm Chemical Reagent Co., Ltd. (Shanghai, China). Acac (AR) and DHB (HPLC) were supplied by Shanghai Myrell Chemical Technology Co., Ltd. (Shanghai, China). All other commercially available reagents were used as received.

### 3.2. Synthesis and Cross Linking of Precursors

A series of single-source precursors were synthesized via a one-pot method using MCl_x_ as the metal source, Acac as a chelating ligand, DHB as a bridging carbon source, and anhydrous ethanol as the solvent. Due to the high sensitivity of MCl_x_ to moisture and oxygen, the synthesis of the precursors was carried out using the standard Schlenk technique [73] under a N_2_ atmosphere with a purity of 99.999%. The synthesis of TaDA is described below as a representative example. Typically, 2.00 g of TaCl_5_ and 1.91 g of Acac were stirred at room temperature for 1 h, yielding a homogeneous orange-yellow solution. Subsequently, 4 mL of anhydrous ethanol was added, and the reaction was continued at 60 °C for 1 h. Then, 0.49 g of DHB (dissolved in 1 mL of anhydrous ethanol) was added, and the obtained mixture was further stirred at 60 °C for 2 h, affording a dark-brown clear solution. The solvent was then removed via reduced-pressure distillation, yielding a light-yellow solid, namely the single-source precursor TaDA. The resulting powders were then cross-linked in a tube furnace under an Ar atmosphere, heated to 200 °C at a rate of 5 °C/min, and held for 1 h, yielding an orange solid. The other precursors were synthesized following the same procedure, with only the metal chloride source and the corresponding molar ratios being varied (Table 1).

### 3.3. Polymer-to-Ceramic Transformation

**Figure 11:**
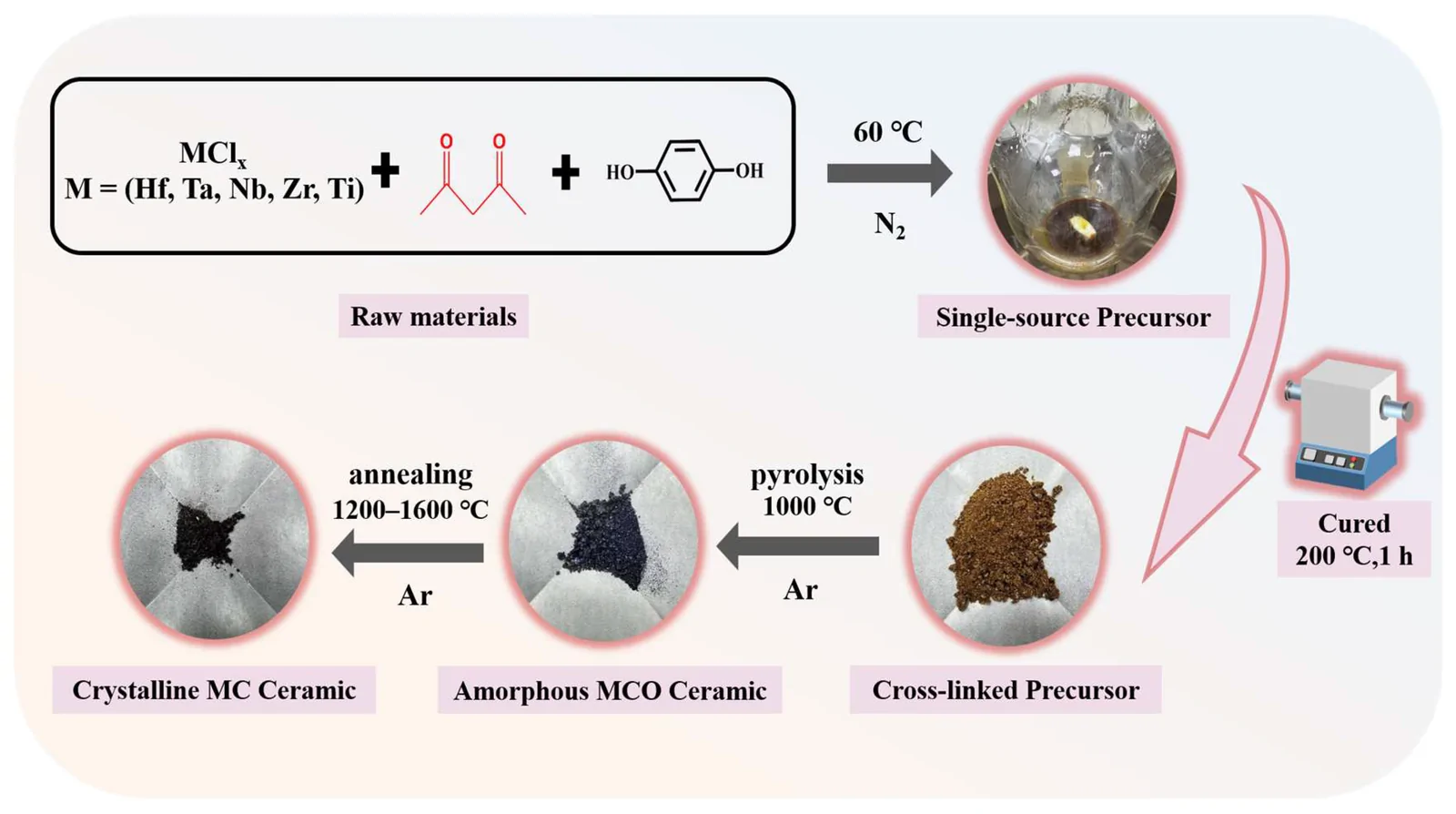
Preparation process of ceramic powders from single-source precursors.

The pyrolysis and annealing processes were conducted in a tube furnace under an Ar atmosphere with a purity of 99.999%. Initially, portions of the cross-linked precursors were heated to 300, 600, 900, and 1000 °C at a rate of 5 °C/min, held for 1 h at each temperature, and then taken out after cooling to room temperature to investigate their polymer-to-ceramic transformation behavior. Then, portions of the ceramic powders obtained after pyrolysis at 1000 °C were heated to 1100, 1200, 1300, 1400, 1450, 1500, and 1600 °C at a rate of 4 °C/min, held for 2 h, and then taken out after cooling to room temperature to study the phase evolution at elevated temperatures. The complete preparation process from the single-source precursor to ceramic powders is illustrated in Figure 11.

### 3.4. Characterization

The molecular structures of the single-source precursors and the ceramic powders were characterized using a Fourier transform attenuated-total-reflection infrared spectrometer (ATR-FTIR, Nicolet iS20, Thermo Fisher, Waltham, MA, USA) with 32 scans, a resolution of 4 cm^−1^, and a wavenumber range of 4000–550 cm^−1^. The thermal behavior and ceramic yield of the cross-linked single-source precursors were analyzed by thermogravimetric analysis (TGA, STA 449 F5 Jupiter, NETZSCH, Selb, Germany) under an Ar atmosphere at a heating rate of 10 °C/min from room temperature to 1400 °C. The phase composition of the ceramic samples was examined by X-ray diffraction (XRD, Ultima IV, Rigaku, Akishima City, Japan) operated at 40 kV and 30 mA with Cu/Kα radiation, over a scanning range of 10–90° (2θ) at 10°/min.

## 4. Conclusions

In summary, a series of single-source precursors (denoted as MDA, M = Hf, Ta, Nb, Zr, Ti) were synthesized via a straightforward one-pot synthesis strategy using MCl_x_ as raw materials, with Acac serving as a chelating ligand and DHB as a bridging carbon source. The as-synthesized precursors are readily soluble in common organic solvents, such as ethanol, a low-cost and non-toxic green solvent, thereby making them suitable as impregnating agents for the fabrication of UHTC-modified CMCs via the PIP approach. The ceramic yields at 1400 °C are 62.45% (TaDA), 57.53% (HfDA), 48.55% (ZrDA), 45.53% (NbDA), and 30.32% (TiDA), and the resulting ceramics exhibit grain sizes in the range of approximately 80–100 nm after heat treatment at their respective transformation temperatures. The oxide-to-carbide phase-transformation temperatures of carbothermal reduction follow the order T_NbC_ (1200 °C) < T_TaC_ (1400 °C) < T_TiC_ (1450 °C) < T_HfC_ (1500 °C) < T_ZrC_ (1600 °C). The mechanism underlying the different phase-transformation temperatures of the MDA-derived ceramics was elucidated through combined thermodynamic and kinetic analyses.

This synthesis strategy shows considerable promise for application to the family of ultra-high temperature refractory metal carbides with melting points exceeding 3000 °C, demonstrating good universality and process adaptability. Due to the straightforward synthesis, these precursors are promising candidates for the preparation of UHTC-based composite materials by the PIP method, providing a theoretical basis for the on-demand design of desired carbides tailored to specific requirements, and offering a versatile route toward practical applications in thermal protection systems for next-generation hypersonic vehicles.

## Figures and Tables

**Table 1:** Molar ratios in the synthesis of MDA precursor.

Sample	MCl_x_ (mol)	DHB (mol)	Acac (mol)
TaDA	1.25	1	4.25
NbDA	1.25	1	4.25
HfDA	1.25	1	3
ZrDA	1.25	1	3
TiDA	1.25	1	3

## Data Availability

Under reasonable request, the data from this work are available from the corresponding author. The data are not publicly available due to institutional restrictions.
